# Skin autofluorescence as a novel marker of vascular damage in children and adolescents with chronic kidney disease

**DOI:** 10.1007/s00467-014-2997-y

**Published:** 2014-11-20

**Authors:** Irena Makulska, Maria Szczepańska, Dorota Drożdż, Dorota Polak-Jonkisz, Danuta Zwolińska

**Affiliations:** 1 Department of Pediatric Nephrology, Wrocław Medical University, ul. Borowska 213, 50-556 Wrocław, Poland; 2 Department of Pediatrics in Zabrze, Medical University of Silesia, Katowice, Poland; 3 Dialysis Unit, Jagiellonian University Medical College, Krakow, Poland

**Keywords:** Chronic kidney disease, Skin autofluorescence, Children, Dialysis, Intima-media thickness, Markers of endothelial damage

## Abstract

**Background:**

Skin autofluorescence (sAF) was examined as a marker of the accumulation of advanced glycation end products (AGEs) in tissues of children with chronic kidney disease (CKD) in relation to renal function, dialysis modality and markers of endothelial inflammation and dysfunction.

**Methods:**

A total of 76 children with CKD were enrolled in the study, of whom 20 children were on hemodialysis (HD), 20 were on peritoneal dialysis (PD) and 36 were treated conservatively. A control group of 26 healthy subjects was also included in the study. In all children, sAF intensity, carotid intima-media (cIMT) thickness and plasma concentrations of sE-selectin, matrix metalloproteinase 9 (MMP-9), tissue inhibitor of metalloproteinase 1 (TIMP-1), asymmetric dimethylarginine (ADMA), symmetric dimethylarginine (SDMA) and plasminogen activator inhibitor type 1 (PAI-1) were measured.

**Results:**

Compared to the controls, children with CKD had significantly elevated sAF levels. sAF in the children with CKD was positively correlated with sE-selectin, MMP-9, TIMP-1, ADMA, SDMA and PAI-1 levels. In the predialysis group (conservative treatment) sAF levels were positively correlated with sE-selectin and ADMA levels and negatively correlated with glomerular filtration rate. Multiple regression analysis showed a significant association of sAF with sE-selectin and MMP-9 in CKD children.

**Conclusions:**

The results reveal that AGEs were accumulated in the children with CKD. This accumulation was related to early vascular changes and a number of biochemical vascular risk markers. sAF measurement, as a noninvasive method, may be useful for identification of clinical risk factors of vascular disease in CKD children.

## Introduction

Cardiovascular complications, including atherosclerosis, are the major cause of morbidity and mortality in patients with chronic kidney disease (CKD) [1], especially in those on maintenance dialysis [2].

 The generation of advanced glycation end products (AGEs) is increased in patients with CKD, which contributes to vascular injury, irrespective of traditional risk factors, such as hypertension, lipid and mineral disorders, smoking and diabetes [3]. AGEs are formed and accumulate as a result of increased oxidative and carbonyl stress and due to reduced renal excretion of their precursors [4]. It has been shown that the increased generation of oxygen free radicals and carbonyl compounds in patients with CKD already occurs in the early stages of the disease and is exacerbated along with its progression [5–7]. The accumulation of AGEs in atherosclerotic plaques which form in the arterial wall of patients with end-stage kidney disease (ESKD) indicates the participation of AGEs in the development of atherosclerotic lesions [8]. The presence of lipoproteins bound to AGEs may increase deposits of oxidized low-density lipoprotein (LDL)-cholesterol in blood vessels, which in turn is associated with the impaired clearance of LDL by a specific receptor [9]. Recent studies have confirmed that AGEs may also play a significant role in the pathogenesis of atherosclerosis through their ability to induce the inflammatory reaction via activation of the receptor for AGEs (RAGE). This receptor is found on various cells, including monocytes, macrophages, vascular endothelial cells and muscle cells. Stimulation of RAGE has been found to increase the expression of adhesion molecules and pro-inflammatory cytokines and stimulate intimal thickening of the arteries and activation of nuclear factor kappa-B, which results in the activation of the inflammatory response and causes stimulation of the host immune system [10, 11]. RAGE receptors located on the vascular endothelium are a crucial factor leading to endothelial dysfunction [10]. The accumulation of AGEs in vascular tissue has a positive correlation with cardiovascular complications and impaired diastolic heart muscle function in adult patients [12].

 It has recently become possible to non-invasively examine the level of AGEs in tissues using the measurement of skin autofluorescence (sAF). Ueno et al. [13] recently confirmed the usefulness of sAF as a predictive marker of the AGE level in tissues by demonstrating a high correlation between sAF and specific AGEs in skin homogenate slices. In a previous study by our research group involving children and adolescents with CKD, we showed for the first time that sAF level is significantly positively correlated with indices of cardio-vascular system damage, such as the index of left ventricular mass and arterial stiffness, based on the alteration in the measurement of pulse wave velocity in the aorta [14]. Elevated values of sAF have been found in patients with acute myocardial infarction and stable coronary artery disease and in adult patients treated with hemodialysis [11]. A high sAF level has been recognized as a predictive factor of increased cardiovascular mortality in adult patients with diabetes and ESKD [15]. Hartog et al. confirmed that increased sAF is an independent predictor of graft function loss in patients who have undergone kidney transplantation [16]. Some authors, including those just mentioned, have concluded that the measurement of sAF can be considered to be a reliable marker of the intensity of inflammation, oxidative stress and atherosclerosis in diseases that generate early complications of the cardiovascular system.

The aim of the study presented here was to evaluate the role of sAF in identifying early vascular changes leading to the development of premature atherosclerosis in children and adolescents with CKD at different stages of the disease. We determined the correlations between sAF and carotid intima-media thickness (cIMT) and the so-called new risk markers for atherosclerosis.

## Patients and methods

### Patients

Seventy-six children with CKD, yet asymptomatic in terms of cardiovascular complications, were enrolled in the study. The patients were divided into three groups according to CKD stage 2–4 [predialysis (Pre) group] and dialysis modality [peritoneal dialysis (PD) and hemodialysis (HD) groups, respectively]. The control group comprised 26 age-matched children with primary nocturnal enuresis and normal kidney function. The clinical and demographic details of the study subjects are summarized in Table 1.

**Table 1:** Clinical and biochemical characteristics of the investigated chronic kidney disease groups and controls

Group parameter	PD (*n* = 20)	HD (*n* = 20)	Pre (*n* = 36)	Controls (*n* = 26)	*p*
Age (years)	14.3 ± 2.3	15 ± 3.3	14.9 ± 3.5	14.5 ± 3.3	NS
Gender (male/female)	12/8	10/10	17/19	12/14	NS
Dialysis vintage (months)	12 ± 11	19 ± 16	–	–	0.02
Body mass index (kg/m^2^)	18.7 ± 3.9	18.7 ± 3.4	20.6 ± 4.1	18.8 ± 3.9	NS
SBP (mm Hg)	117 ± 11^a,c^	128 ± 13^a,b,d^	115 ± 10^a^	100 ± 9	<0.0001
DBP (mm Hg)	75 ± 11^a,c^	82 ± 10^a,b,d^	71 ± 8^a^	64 ± 6	<0.0001

Values are presented as the mean ± standard deviation (SD) or as a number, where appropriate

PD, peritoneal dialysis; HD, hemodialysis; Pre, predialysis group—conservative treatment; SBP, systolic blood pressure; DBP, diastolic blood pressure; NS, non significant

^a^Versus control

^b^Versus PD

^c^Versus HD

^d^Versus Pre

 The PD group included 20 children on PD, with 12 patients on night intermittent PD and eight patients on continuous cyclic PD in eight patients. For all PD patients, the HomeChoice automated PD system was used (Baxter International, Inc., Deerfield, IL). Standard dialysis solutions with 1.5 and 2.3 % glucose concentration and 1.25 or 1.75 mmol/l calcium concentration were applied according to individual recommendations. The causes of CKD in children in the PD group included structural urinary tract abnormalities (9 patients), glomerulonephritis (5), polycystic kidney disease (3), hereditary nephropathy (2) and hemolytic uremic syndrome (1). Fifteen children remained on hypotensive medication, which included angiotensin-converting enzyme inhibitors (ACEi) (13 patients), calcium channel blockers (6) and β-blockers (3). All patients received calcium-containing phosphate binders and vitamin D metabolites in doses adjusted to current requirements.

The HD group included 20 children on maintenance HD. Dialysis sessions were performed three times a week (3–5 h) using polysulfone membranes**.** Blood flow ranged from 120 to 250 ml/min, and dialysate flow did not exceed 500 ml/min. Dialysis fluid was buffered with bicarbonate and contained 1.25 or 1.5 mmol/l calcium. The causes of CKD in this group were urinary tract abnormalities (CAKUT) (12 patients), glomerulonephritis (6) and hereditary glomerulopathy (2). Nineteen hypertensive children were treated with ACEi (19 patients), calcium channel blockers (11) and/or β-blockers (2) patients. All HD patients received calcium-containing phosphate binders and vitamin D compounds.

The Pre group included 36 children with CKD stage 2–4 on conservative treatment. The causes of CKD in this group were urinary tract malformations (22 patients), glomerulonephritis (5), polycystic kidney disease (3), hereditary glomerulopathy (2), unknown cause(s) (2), hemolytic uremic syndrome (1) and complications after chemotherapy for cancer (1). Stage 2 CKD was detected in 13 children, stage 3 in ten children and stage 4 in 13 children. Twelve patients were treated with ACE-I, six were treated with angiotensin receptor blockers, four received calcium channel blockers and one received a β-blocker.

All patients with CKD stage 3 and 4, and five children with stage 2 received treatment with calcium-containing phosphate binders and vitamin D compounds.

Children under 6 years of age and patients with diabetes and infection were excluded from the study. Informed consent for participation in the study was obtained from all the parents and children over the age of 15 years. The research project was approved by the Wroclaw Medical University ethics committee.

### Methods

Progression of CKD was evaluated in accordance with the Kidney Disease Outcomes Quality Initiative (K/DOQI) guidelines published in 2002 [17]. Estimated glomerular filtration rate (GFR) was determined by the Schwartz formula [18]. All children underwent the following examinations: serum creatinine, blood pressure measurement (BP), sAF assessment, carotid intima-media thickness (cIMT) evaluation and body mass index (BMI) determination. Blood samples obtained after overnight fasting were drawn from the peripheral vein in patients from the PD, Pre and control groups and from the afferent line prior to starting an HD session in HD children. Biochemical tests on serum samples included creatinine level estimation. The parameters were measured according to automated standardized laboratory techniques in the clinical laboratory using a multichannel analyzer (model KONELAB30i; Thermo Fisher Scientific Inc., bioMérieux, Marcy l'Etoile, France). Blood for the evaluation of endothelial markers was collected in dry tubes and subsequently centrifuged (3,000 *g*, 15 min); the serum was frozen and stored at −20 °C until assayed. Blood for plasminogen activator inhibitor-1 (PAI-1) measurement was collected in EDTA tubes and centrifuged (1,000 *g* for 15 min) within 30 min of collection. After separation from blood cells, the plasma was frozen and stored at −20 °C until assayed.

sE-selectin, asymmetric dimethylarginine (ADMA), symmetric dimethylarginine (SDMA), matrix metalloproteinase 9 (MMP-9), tissue inhibitor of metalloproteinase 1 (TIMP-1) and PAI-1 levels, respectively, were determined twice, and the average values were used for analysis. sE-selectin/CD62E was assessed using a commercially available enzyme-linked immunosorbent assay (ELISA) kit (R&D Systems, Inc. Minneapolis, MN) according to the manufacturer’s instructions. Commercially available ELISA kits were also used to determine ADMA (Immundiagnostik AG, Bensheim, Germany), TIMP-1 and PAI-1/Human Serpin E1 (ELISA kits; R&D Systems). SDMA was determined using a DLD Diagnostika GmbH kit (Hamburg, Germany), and MMP-9 was determined using a kit produced by R&D Systems, Inc. 

Both weight and height were measured using standardized protocols. BMI was calculated as weight in kilograms divided by height in meters squared.

BP measurements with the oscillometric device were performed according to the recommendations of the Fourth Report of the Blood Pressure Control in Children Working Group [19].

#### Skin autofluorescence

Skin autofluorescence was assessed using the AGE Reader device (Diagnoptics BV, Groningen, The Netherlands) as described previously in detail by Meerwald et al. [20]. This is a noninvasive device used to assess the accumulation of fluorescent AGEs in tissues. In brief, the ratio of the average light intensity per nanometer in the range between 420 to 600 nm emitted by the source divided by the average of excited light intensity per nanometer in the range between 300 and 420 nm was used as the measurement of autofluorescence. The intra- and inter-day coefficient of variation for autofluorescence reader measurements was 2.7 % (*n* = 8) and <6 % (*n* = 10), respectively. sAF was expressed in arbitrary units (AU). All measurements were performed on normal skin of the lower arm (ventral site, about 5 cm distal to the antecubital space), without any abnormalities, at room temperature, with the patient in a seated position.

#### Carotid intima-media thickness

Ultrasonographic examinations of intima-media thickness of both common carotid arteries (cIMT) were performed using the ALOKA ProSound SSD-5500 SV type ultrasound system equipped with vascular 7–12 MHz probe (Hitachi Ltd., Tokyo). All measurements were made by the same investigator, who was blinded to the medical history of the patient being evaluated. During the examination the child was in the supine position with his/her head slightly tilted in the opposite direction to the area being tested. cIMT measurements were made on the back wall of both common carotid arteries, at a height of 1–2 cm below the bifurcation. The distance between two distinctly shining lines of the back wall was assessed. The first shining line represents the border between the vessel and the inner membrane, and the second one represents the border between the middle membrane and adventitia. The measurements were repeated four times, and the mean value from all the tests was regarded as the final result.

### Statistical analysis

The results were expressed as mean values ± standard deviation when a normal distribution of variables was obtained. The differences were then compared by analysis of variance (ANOVA) tests. In the case of a non-normal distribution, a non-parametric Kruskal–Wallis test for median values was used. For evaluation of the relationship between factors, Pearson’s correlation test was performed when a normal distribution of variables was obtained, and Spearman’s test was applied for data with a non-normal distribution. Multiple regression analysis was performed with sAF as a dependent variable. Since the* p* value in the ANOVA table was <0.05, there was a statistically significant relationship between the variables at the 95.0 % confidence level. Statistical analyses were performed using the package STATGRAPHICS (Centurion XV v.15.2.06; Statpoint Technologies, Warrenton, VA). A* p* value of <0.05 was considered to be statistically significant.

## Results

The clinical and biochemical characteristics of the investigated patients are described in Table 1. The groups were matched for age, gender and BMI values. The highest values of systolic BP (SBP) and diastolic BP were observed in children on HD, and these were significantly different from those of children in the PD, Pre and control groups. The mean time on PD was shorter than that of maintenance HD duration.

Table 2 shows the results of the sAF, cIMT and endothelial marker measurements in the examined groups with two-by-two comparison.

**Table 2:** Measured values of sAF, cIMT, sE-selectin, ADMA, SDMA, MMP-9, TIMP-1 and PAI-1 in patient groups and control group

Group parameter	PD (*n* = 20)	HD (*n* = 20)	Pre (*n* = 36)	Controls (*n* = 26)	*p*
sAF (10^−2^ AU)	2.46 ± 0.72^a,d^	2.61 ± 0.57^a,d^	1.9 ± 0.46^a,b,c^	1.33 ± 0.26	<0.0001
cIMT (left carotoid artery) (mm)	0.41 ± 0.05^a,c^	0.45 ± 0.04^a,b,d^	0.41 ± 0.04^a,c^	0.37 ± 0.04	<0.0001
cIMT (right carotoid artery) (mm)	0.41 ± 0.05^a,c^	0.44 ± 0.04^a,b,d^	0.41 ± 0.04^a,c^	0.36 ± 0.03	<0.0001
sE-selectin (ng/ml)	53.34 ± 0.49^a,c,d^	66.32 ± 1.59^a,b,d^	46.34 ± 0.94^a,b,c^	24.05 ± 0.48	<0.0001
ADMA (μmol/l)	0.78 ± 0.005^a,c,d^	0.85 ± 0.01^a,b,d^	0.65 ± 0.03^a,b,c^	0.39 ± 0.008	<0.0001
SDMA (μmol/l)	0.79 ± 0.009^a,c,d^	0.94 ± 0.01^a,b,d^	0.69 ± 0.02^a,b,c^	0.48 ± 0.006	<0.0001
MMP-9 (ng/ml)	642 ± 4.42^a,c,d^	700.7 ± 5.29^a,b,d^	430 ± 7.35^a,b,c^	108.9 ± 3.35	<0.0001
TIMP-1 (ng/ml)	157.71 ± 3.73^a,c,d^	415.7 ± 28.52^a,b,d^	133.76 ± 2.75^a,b,c^	83.13 ± 1.67	<0.0001
PAI-1 (ng/ml)	15.83 ± 0.28^a,c,d^	17.65 ± 0.64^a,b,d^	11.38 ± 0.43^a,b,c^	6.27 ± 0.26	<0.0001

Values are presented as the mean ± SD

sAF, Skin autofluorescence; cIMT, Carotid intima-media thickness; ADMA, Asymmetric dimethylarginine; SDMA, symmetric dimethylarginine; MMP-9, matrix metalloproteinase-9; TIMP-1, tissue-1 inhibitor metalloproteinases; PAI-1, plasminogen activator inhibitor type 1; AU arbitrary units

^a^Versus control

^b^Versus PD

^c^Versus HD

^d^Versus Pre

sAF was significantly higher in all groups of children with CKD than in the controls, with the highest sAF values observed in the HD group. However, we were unable to demonstrate a significant difference in sAF between the HD group and the PD group. The highest values of cIMT were observed in children in the HD group, and these were significantly different from the cIMT values of the other groups. cIMT values were also increased in the PD and Pre groups as compared to controls. All examined endothelial marker values were highest in the HD patients. Endothelial marker values of children in the PD and Pre groups were also significantly increased compared to those of the controls.

### Analysis of factors associated with sAF

**Fig. 1:**
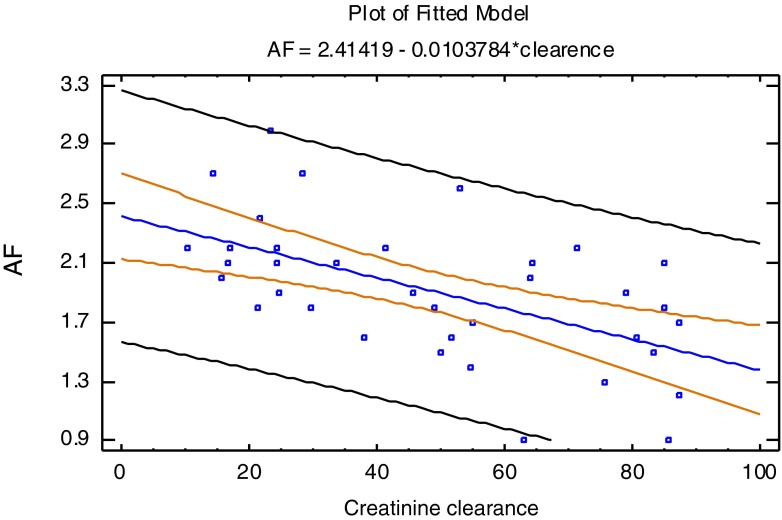
Correlation between sAF and creatinine clearance in predialysis children (*r* = −0.37; *p* = 0.0003). sAF - skin autofluorescence (10^-2^ AU), Creatinine clearance (ml/min/1.73 m^2^)

Significantly positive linear correlations between sAF and the mean levels of sE-selectin, MMP-9, TIMP-1, ADMA, SDMA and PAI-1, respectively, were observed in all of the CKD children (PD, HD and Pre groups) (Table 3). In the Pre group, sAF level was positively correlated with sE-selectin and ADMA levels, respectively, and negatively correlated with creatinine clearance (*p* = 0.0003). Detailed data are shown in Table 3 and Fig. 1.

**Table 3:** Analyses of factors correlated with skin autofluorescence (10^−2^ AU)

Variable	Pearson correlation test	
	*r*	*p*
sAF (10^−2^ AU) (PD + HD + Pre)		
MMP-9 (ng/ml)	0.45	<0.0001
TIMP-1 (ng/ml)	0.23	0.04
sE-selectin (ng/ml)	0.46	<0.0001
ADMA (μmol/l)	0.41	<0.0001
SDMA (μmol/l)	0.37	<0.0001
PAI-1 (ng/ml)	0.46	<0.0001
sAF (10^−2^ AU) (Pre)		
sE-selectin (ng/ml)	0.42	0.01
ADMA (μmol/l)	0.35	0.03
Creatinine clearance (ml/min/1.73 m^2^)	−0.37	0.0003

sAF, Skin autofluorescence; cIMT, Carotid intima-media thickness; ADMA, Asymmetric dimethylarginine; SDMA, symmetric dimethylarginine; MMP-9, matrix metalloproteinase-9; TIMP-1, tissue-1 inhibitor metalloproteinases; PAI-1, plasminogen activator inhibitor type 1; AU arbitrary units; PD, peritoneal dialysis; HD, hemodialysis

A multiple regression analysis of all children with CKD revealed significantly positive associations between sAF and sE-selectin and MMP-9 levels, respectively, as shown in Table 4. Also in the entire CKD group, the independent predictors of cIMT were age, SBP and sE-selectin concentration (Table 5).

**Table 4:** Multiple regression analysis of factors associated with skin autofluorescence

Variable	*R* ^2^	β	95 % CI	*p *
sAF (10^−2^ AU) (PD + HD + Pre)				
MMP-9 (ng/ml)	29.6 %	0.007	−0.01-0.004	<0.0001
sE-selectin (ng/ml)	29.6 %	0.07	−0.01- 0.17	0.03

CI, Confidence interval; MMP-9, matrix metalloproteinase; AU, arbitrary units; sAF, skin autofluorescence; PD, peritoneal dialysis; HD, hemodialysis

**Table 5:** Multiple regression analysis of factors associated with carotid intima-media thickness

Variable	*R* ^2^	β	95%CI	*p*
cIMT (mm) (PD+HD+PRE)				
Age (years)	33.1 %	0.006	0.003–0.01	0.006
SBP (mm Hg)	33.1 %	0.0005	−0.002 to 0.001	0.0005
sE-selectin (ng/ml)	33.1 %	0.0007	−0.0008 to 0.002	0.0007

cIMT, Carotid intima-media thickness; SBP, systolic blood pressure; PD, peritoneal dialysis; HD, hemodialysis

## Discussion

Recent studies indicate that AGEs play a significant role in the development of atherosclerotic lesions [21, 22]. AGEs are classified as uremic toxins and may be formed in the course of hyperglycemia, hyperlipidemia and oxidative and carbonyl stress. Patients with ESKD are especially vulnerable to the accumulation of AGEs: (1) they are at a higher risk of enhanced formation of AGEs “de novo” as a result of the increased oxidative stress which accompanies uremia; (2) they experience decreased and eventually stopped renal excretion of these molecules’ precursors [23]. Recent research has confirmed that AGEs may also play a significant role in the pathogenesis of atherosclerosis due to their ability to induce the inflammatory reaction by activation of RAGE [24]. In the last decade, noninvasive examination of the levels of AGEs in tissues based on sAF measurements has become possible. In our study we report, for the first time, the association between sAF and various risk factors of endothelial dysfunction and the development of atherosclerosis in children with CKD. We found that sAF values were elevated in all stages of the disease. We also assessed correlations of sAF with traditional and “new” atherosclerosis risk factors, such as the cIMT and the concentration of markers of inflammation and/or endothelial damage, including sE-selectin, MMP-9, TIMP-1, ADMA, SDMA and PAI-1. Compared to the healthy control group, elevated sAF values were found in all three groups of CKD patients. As expected, the highest sAF values were observed in children undergoing HD, although these did not differ significantly from the values obtained in children on PD. These results are consistent with those reported from studies in adults [16, 25, 26]. Meerwald et al. [26] examined 109 patients treated with HD and showed that the sAF level in these patients was twofold higher that in healthy subjects. These authors also found that sAF intensity was an independent predictor of increased cardiovascular mortality. In turn, Hartog et al. [27] demonstrated, independently of other factors, the relationship of sAF with diastolic myocardial function, which is a frequently observed impairment in adult patients with ESKD. One of the few studies in the pediatric population focused on the concentrations of AGEs in plasma. In this study, Sebekova and colleagues [28] evaluated the concentrations of AGEs [fluorescent molecules and carboxymethyllysin (CML)] in children and adolescents with CKD, among which 18 subjects were treated conservatively, ten remained on maintenance dialysis and nine received kidney transplantation. All patients showed elevated concentrations of AGEs, with the patients on dialysis having the highest values. There was no significant correlation between the levels of AGE in the serum and markers of inflammation [C-reactive protein (CRP), interleukin-6, tumor necrosis factor-α]. Although these results are similar to these obtained in our evaluation of sAF, they may not be interpreted similarly. In the study of Sebekova and colleagues [28] there was no evidence for a close correlation between sAF and circulating AGEs, except for CML, which is only one of numerous AGEs [29]. In our study we demonstrated a positive correlation between sAF and cIMT in the entire study population. cIMT measurement is a well-known and recognized noninvasive method of assessing the degree of severity of atherosclerotic lesions, and the validity of this diagnostic marker has been confirmed by many researchers, including Nishizawa et al. [30] and Tanaka et al. [31] in adult patients treated with maintenance hemodialysis. The authors of the latter study proved that in a population of 128 dialyzed adults, cIMT, high-sensitivity CRP and sAF were independent predictors of cardiovascular complications. In our previous study we also demonstrated a relationship between sAF and the duration of dialysis and BP values, which is consistent with the observations of Japanese researchers [14, 22]. In the present study, we have shown for the first time that in a population of children with CKD, there is a relationship between sAF measurements and endothelial markers such as sE-selectin, PAI-1, MMP-9, TIMP-1, ADMA and SDMA. The significantly higher concentrations of the latter compounds we found our pediatric patients with CKD indicate that the stimulation and/or endothelial damage, which is the first step in the process of atherogenesis, is already present in the predialytic period and subsequently increases in ESKD. Previous studies have shown conclusively that chronic inflammation and oxidative stress play an important role in the pathogenesis of atherosclerosis. A significant involvement of sE-selectin in the process of atherogenesis was suggested by Testa et al. [32], who demonstrated that the Leu554Phe polymorphism of the gene encoding E-selectin is a prerequisite for the occurrence of severe atherosclerotic lesions. To date, few studies have shown that this mechanism also occurs in children with CKD [33, 34]. We report here for the first time that we found a positive correlation between sAF measurements and sE-selectin concentration in children with CKD. Recent studies indicate an important role of the action of MMPs and their inhibitors in vascular remodeling in the pathogenesis of atherosclerosis. Clinical studies have shown that the imbalance between MMP-9 and its inhibitor TIMP-1 plays a significant role in the development of cardiovascular pathology [35–39]. In our study we demonstrated, for the first time, a positive correlation between sAF intensity and MMP-9 and TIMP-1 concentrations, respectively, in children with CKD. ADMA and SDMA, as endogenous nitric oxide synthase inhibitors, play an important role in the pathogenesis of cardiovascular disease by decreasing the synthesis of nitric oxide, which is a key vasodilator factor [40, 41]. We demonstrated significant correlations between sAF and ADMA and SDMA levels. The relationship between PAI-1 overexpression and atherogenic changes has been shown in mouse models [42] and in adults without kidney dysfunction [43]. However, regarding the aspect of PAI-1 concentration and cardiovascular complications, there have only been a few studies in adults and none prior to our study in children [44–48]. We documented a positive correlation between PAI-1 and sAF in the entire study group of children with CKD, as well as a positive correlation between sAF and cIMT. In a recent study performed in children with CKD [14], we showed significant correlations between sAF and markers of calcium and phosphate metabolism abnormalities (P concentration, Ca × P index) and hypertension; these correlations support available data on the harmful influence of the accumulation of AGEs in tissues in these disorders as well. It is therefore worth emphasizing that with the progression of CKD in children, there is an increased accumulation of AGEs in tissues, which was confirmed in our previous study [14], where we showed a negative correlation between sAF and GFR. Multivariate regression analysis in our pediatric patient cohort with CKD showed that the independent predictors of sAF were sE-selectin and MMP-9 concentrations, and in the entire population, cIMT. These results confirm that sAF may be used as a marker of the intensity of vascular endothelial damage of inflammatory and atherosclerotic lesions in children with CKD.

Our study has several limitations. Firstly, the sample size, particularly the size of the subgroups, may be insufficient to show significant differences. Our patients were recruited from three of the pediatric nephrology centers which treat children from the southern region of Poland. Future studies with a larger sample size are necessary, especially in the light of new therapeutic methods which enable the lowering of AGEs. Animal experiments have documented that some AGE breakers may prevent alterations in vessels [49]. Lewis et al. suggests that patients with less advanced stages of CKD might benefit by receiving pyridoxamine, which may inhibit the formation of AGEs [50].

## Summary

The results obtained in our study indicate a link between the accumulation of AGEs in tissues, measured as sAF intensity, and vascular inflammatory and atherosclerotic changes in children and young adults with CKD. We therefore suggest that sAF may be, in the future, a useful tool for risk stratification in this group of patients and an easy and repeatable method for monitoring the effects of therapy. Further studies are needed to clarify whether the accumulation of AGEs in tissues could be a new therapeutic target for improving cardiovascular complications in the CKD population.
